# Racial and Ethnic Concordance Between National Health Service Corps Clinicians and Underserved Populations

**DOI:** 10.1001/jamanetworkopen.2024.2961

**Published:** 2024-03-20

**Authors:** Olesya Baker, Marcela Horvitz-Lennon, Hao Yu

**Affiliations:** 1 Department of Population Medicine, Harvard Medical School and Harvard Pilgrim Health Care Institute, Boston, Massachusetts; 2 RAND Corporation, Boston, Massachusetts

## Abstract

**Importance:**

Despite the widely recognized importance of racial and ethnic concordance between patients and clinicians, there is a lack of studies on clinician diversity in medically underserved areas and whether it aligns with the changing demographic landscape.

**Objective:**

To assess trends in National Health Services Corps (NHSC) clinician diversity and racial and ethnic concordance between NHSC clinicians and the populations in underserved areas from before to after the 2009 NHSC expansion.

**Design, Setting, and Participants:**

This cross-sectional, population-based study compared trends in the diversity of NHSC clinicians practicing in health professional shortage areas (HPSAs) and the HPSA populations during 2003 to 2019 using the Health Resources and Services Administration’s NHSC Field Strength Database and Area Health Resources Files. The analysis was performed from February through May 2023.

**Main Outcomes and Measures:**

Concordance was measured with an annual community representativeness ratio defined as the ratio of the proportions of same race or ethnicity NHSC clinicians to HPSA population.

**Results:**

There were a total of 41 180 clinicians practicing in HPSAs from 2003 to 2019; the median (IQR) age was 34 (30-41) years. Among 38 569 NHSC clinicians who reported gender, 28 444 (73.7%) identified as female and 10 125 (26.3%) identified as male. The average annual number of NHSC clinicians increased from 3357 in 2003 to 2008 to 9592 in 2009 to 2019. Before 2009, 1076 clinicians (5.3%) identified as Black, 9780 (48.6%) as Hispanic, 908 (4.5%) as other, and 8380 (41.6%) as White. During this period, concordance was low among non-Hispanic White and Black individuals due to clinician underrepresentation relative to the population, yet Hispanic clinicians were overrepresented. Following the 2009 NHSC expansion, the main change was the sharp decline in the proportion of Hispanic clinicians, to 1601 (13%) by 2019; while concordance was achieved for non-Hispanic White and Black individuals, Hispanic clinicians became underrepresented relative to population. The results held across 3 specialties: primary care, mental health care, and dental care.

**Conclusions and Relevance:**

This cross-sectional study of trends in racial and ethnic concordance found that while the NHSC expansion starting in 2009 improved clinician-population concordance for non-Hispanic White and Black individuals, it reversed a prior trend for Hispanic individuals among whom clinicians became underrepresented relative to the population. Targeted NHSC clinician recruitment efforts are needed to improve concordance for Hispanic individuals in underserved areas, especially given Hispanics’ projected growth in the US.

## Key Points

**Question:**

What are the long-term trends in racial and ethnic concordance between National Health Service Corp (NHSC) clinicians and the population of medically underserved areas before and after the 2009 NHSC expansion?

**Findings:**

This cross-sectional study of 41 180 NHSC clinicians found that since the 2009 expansion, NHSC clinician-population concordance was achieved for non-Hispanic White and Black individuals. However, the representation of Hispanic NHSC clinicians changed from overrepresentation to underrepresentation relative to population after 2009.

**Meaning:**

These findings suggest NHSC clinician racial and ethnic composition became more concordant with the racial and ethnic composition of the population in their practice areas, except for Hispanic NHSC clinicians.

## Teaser

This cross-sectional study evaluates trends in National Health Services Corps (NHSC) clinician diversity and racial and ethnic concordance between NHSC clinicians and underserved populations.

## Introduction

The US population has become more diverse in recent years, with substantial growth of Hispanic and non-Hispanic Black populations compared with non-Hispanic Whites.^1,2,3,4,5^ The US Census Bureau projects a further decline in the number of non-Hispanic White individuals and substantial growth in the non-Hispanic Black and Hispanic populations by 2050.^6^

As the US population changes, clinicians are caring for an increasingly diverse patient population.^6^ Changes in the racial and ethnic composition of the patient population present clinicians with unique challenges, such as cultural and language barriers, which can impact the delivery of effective and equal care. Studies have demonstrated that patient-clinician racial and ethnic concordance can improve patient care and health outcomes and reduce health expenditures.^7,8,9,10,11,12,13,14^ Several professional organizations have issued statements promoting clinician diversity and supporting concordant care.^15,16,17,18^ Providing racially and ethnically concordant care may be particularly challenging in areas with health workforce shortages, which make it more difficult for patients to find racially and ethnically concordant clinicians.^19,20,21^ However, the existing literature provides limited insights regarding trends in clinician diversity in underserved areas and how they align with the changing demographic landscape.

The National Health Service Corps (NHSC) program addresses health professional shortages by providing loan repayment and scholarships to clinicians who commit to working in health professional shortage areas (HPSAs). There are 3 types of HPSAs designated by the Health Resources and Services Administration (HRSA): primary care, mental health care, and dental care. Each of them is eligible for recruiting NHSC clinicians. Since 2009, there has been a significant increase in NHSC funding. Its annual budget was initially more than doubled under the 2009 American Recovery and Reinvestment Act,^22,23^ and then further increased under the 2010 Patient Protection and Affordable Care Act, which permanently reauthorized the NHSC.^22^ As a result, the primary care workforce employed by the NHSC program increased by 136% between 2009 and 2011,^23,24^ and more than tripled by 2022.^22^

The 2009 NHSC expansion substantially increased the number of NHSC clinicians practicing in underserved areas.^23^ While policymakers acknowledge the NHSC’s potential in diversifying the health workforce in underserved areas,^25^ it remains unclear if the postexpansion NHSC clinician increases were accompanied by racial and ethnic alignment with the population they serve. This study addresses this gap by analyzing trends in NHSC clinician racial and ethnic composition and its concordance with the racial and ethnic composition of the population in their practice areas.

## Methods

In this cross-sectional study, we used the 2003 to 2019 HRSA’s NHSC Field Strength Database, which contained clinician-level information on NHSC program type, specialty, practice site, and self-reported race and ethnicity. The Harvard Pilgrim Health Care Institute institutional review board deemed this study as nonhuman participants research and waived the need for informed consent because the data were deidentified. This study followed the Strengthening the Reporting of Observational Studies in Epidemiology (STROBE) reporting guideline for cross-sectional studies. Our analysis included NHSC clinicians in either the NHSC Loan Repayment or the NHSC Scholarship program, both of which were in place throughout our study period. As a sensitivity check, we also examined each program type separately. We created 4 mutually exclusive race and ethnicity groups: non-Hispanic White (hereafter White), non-Hispanic Black (hereafter Black), non-Hispanic other (American Indian and Alaska Native, Asian, Native Hawaiian and Other Pacific Islander, and other race; hereafter other), and Hispanic. We used clinicians’ specialty to classify NHSC clinicians into 3 specialty domains: primary care, mental health care, and dental care. We first aggregated clinician-level data into annual counts of clinicians stratified by domain and race and ethnicity. To analyze geographic variation within a given year, we also created county-level counts of NHSC clinicians using clinician’s zip code and applying the Department of Housing and Urban Development US Postal Service zip code to county crosswalk file.^26^ In a subgroup analysis we further stratified NHSC clinician counts by clinician type (physicians vs other clinicians; see eTable in Supplement 1 for classification details).

To our knowledge, person-level racial and ethnic information for patients receiving health care from NHSC clinicians is not publicly available. Therefore, we used 2 approaches to identify a likely patient population for NHSC clinicians. First, we considered individuals who may receive care from NHSC clinicians based on their residence in an HPSA-designated county. Specifically, we separately examined 3 types of HPSA designations: primary care, mental health, and dental HPSAs. HPSA designations can apply to the entire county (whole shortage) or at a subcounty level (partial shortage). Because NHSC clinicians can serve in either whole or partial shortage counties, we first analyzed the demographic composition of whole and partial shortage counties combined. Additionally, we investigated HPSA counties that were designated as whole shortage throughout the study period. This subgroup is small, with 40 primary care, 88 mental health care, and 52 dental care HPSAs, because many whole shortage counties were redesignated to partial shortage status under the Shortage Designation Modernization Project that was implemented in 2014 to streamline designations as part of the Patient Protection and Affordable Care Act.^27^ However, because of its persistent whole shortage status, this subgroup represents populations with the most critical need for health care services. We identified counties’ HPSA status and demographic characteristics using the 2003 to 2019 Area Health Resources Files from HRSA. Race and ethnicity of a county population are self-reported by respondents to surveys and censuses by the United States Census Bureau and incorporated into the Area Health Resources Files.

This approach assessed the demographic composition within all counties with HPSA designations. However, the demographic composition of HPSA-designated counties that received NHSC clinicians may differ from those HPSA-designated counties that were eligible to but did not receive any NHSC clinicians in a given year between 2003 to 2019. Therefore, our second approach focused on a subset of HPSA-designated counties with at least 1 NHSC clinician in a given study year.

### Statistical Analysis

We assessed the relative concordance between the NHSC workforce and the patient population using a community representativeness ratio measure, also known as the diversity index.^28,29,30^ As in prior studies, we defined this measure as the ratio of the proportion of NHSC clinicians of a given race or ethnicity divided by the proportion of the patient population who identified as the same race or ethnicity.^28^ The community representativeness ratio is equal to 1 when the proportion of NHSC clinicians of a given race or ethnicity is equal to the proportion of the HPSA-designated population of the same race or ethnicity. We refer to this state as parity. A community representativeness ratio greater than 1 indicates overrepresentation of NHSC clinicians compared with the population, while a community representativeness ratio less than 1 indicates underrepresentation. We computed the community representativeness ratio at the nationwide level using the aggregate data and at the county level using the county-level data.

We introduced an additional county-level concordance measure— clinician-population ratio— defined as the number of county-level NHSC clinicians of a given race or ethnicity divided by the number of county residents who identified as the same race or ethnicity. We developed this measure because in counties with a very small total number of NHSC clinicians, the percentage of NHSC clinicians of a given race and ethnicity often greatly exceeds the percentage of the county population of the same race and ethnicity, causing the county-level community representativeness ratio to become inflated. We described changes in the number and racial and ethnic composition of NHSC clinicians and investigated temporal trends in the community representativeness and clinician-population ratios at the national and county levels, stratified by HPSA designation type. All analyses were conducted using Stata MP version 16 (Stata Corp LLC). Analyses were performed from February through May 2023.

## Results

Among the 41 180 NHSC clinicians practicing in HPSAs during 2003 to 2019 included in the study, the median (IQR) age was 34 (30-41) years. Among 38 569 NHSC clinicians who reported gender, 28 444 (73.7%) identified as female and 10 125 (26.3%) identified as male; the average annual number of NHSC clinicians increased from 3357 in 2003 to 2008 to 9592 in 2009 to 2019. Before the 2009 NHSC expansion, the racial and ethnic composition of NHSC clinicians remained relatively stable with 1076 NHSC clinicians identifying as Black (5.3%), 9780 (48.5%) as Hispanic, 8380 (41.6%) as White, and 908 (4.5%) as other races and ethnicities.

**Figure 1.**
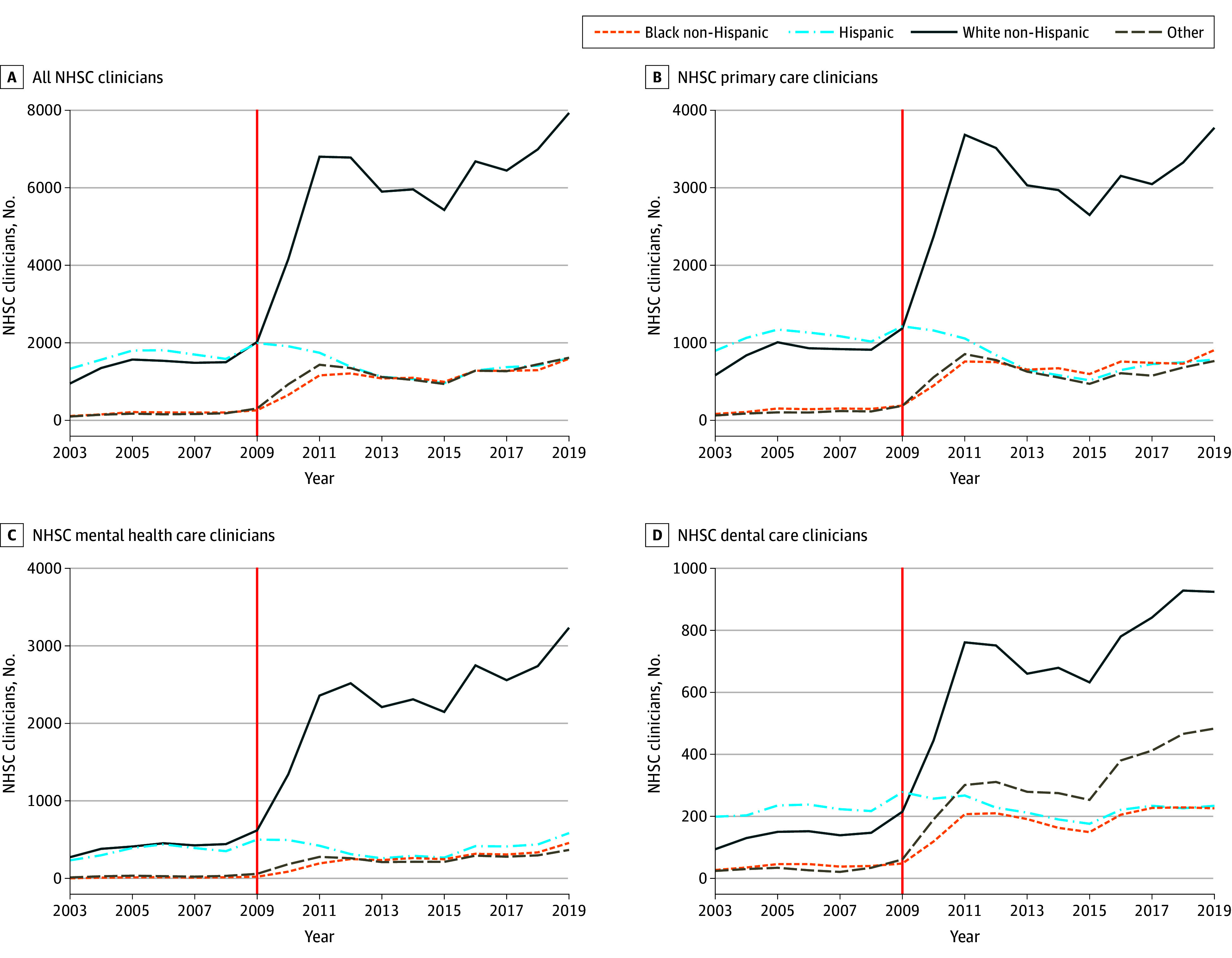
Number of National Health Service Corps (NHSC) Clinicians by Race and Ethnicity, Overall and by Clinician Specialty Vertical line denotes the 2009 NHSC expansion. Race and ethnicity of NHSC clinicians are self-reported in the NHSC clinician database compiled by Health Resources and Services Administration. This study defined 4 categories of race and ethnicity, including Hispanic, non-Hispanic Black, non-Hispanic White, and non-Hispanic other (American Indian and Alaska Native, Asian, Native Hawaiian and Other Pacific Islander, and other race). Source: Authors’ analysis of NHSC Field Strength data, fiscal year 2003 to fiscal year 2019.

Fueled by the 2009 NHSC expansion, there was a steady growth in the number of NHSC clinicians. This growth was primarily due to an increase in White NHSC clinicians, as depicted in Figure 1A. During the postexpansion period, there was a slight decline in the number of Hispanic NHSC clinicians and a sharp decline in their proportional representation relative to the NHSC workforce. By 2019, Hispanic NHSC clinicians accounted for only 13% (1601 clinicians) of the overall NHSC clinician workforce, whereas White, Black, and other clinicians accounted for 62% (7929 clinicians), 12% (1588 clinicians), and 13% (1614 clinicians), respectively. Stratifying by NHSC program type, we observed similar trends among NHSC clinicians enrolled in the loan repayment program and a more pronounced decline among Hispanic NHSC clinicians enrolled in the scholarship program (eFigure 1 in Supplement 1).

Figures 1B, C, and D illustrate temporal trends in the number and the racial and ethnic composition of NHSC clinicians stratified by clinical fields of primary care, mental health care, and dental care, respectively. The trends in each field align with the overall NHSC workforce and diversity trends. In particular, before 2009, the number and racial and ethnic composition of NHSC clinicians remained relatively stable for each field. After the 2009 NHSC expansion, the number of NHSC clinicians significantly increased in each field, primarily due to a rise in White NHSC clinicians. By 2019, White clinicians represented the majority, accounting for 60% in primary care, 70% in mental health care, and 50% in dental care.

**Figure 2.**
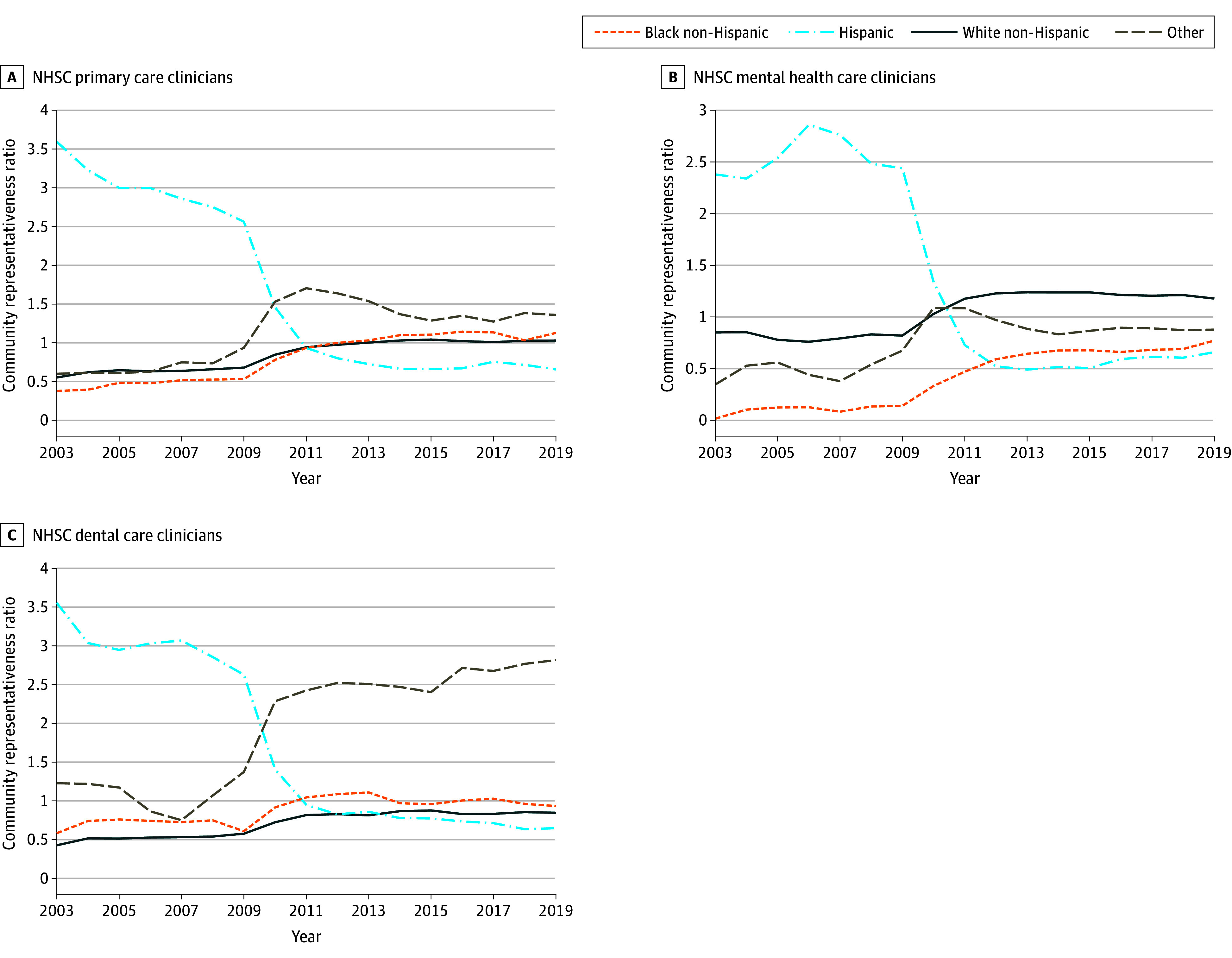
Community Representativeness Ratio by Clinician Specialty and Race and Ethnicity The community representativeness ratio measure was defined as the ratio of the proportion of National Health Service Corps (NHSC) clinicians of a given race and ethnicity divided by the proportion of the population of shortage counties who identified as the same race and ethnicity. Diversity indices were calculated for primary care, mental health care, and dental care NHSC clinicians relative to the population of primary care, mental health care, and dental care health professional shortage areas. Race and ethnicity of NHSC clinicians are self-reported in the NHSC clinician database compiled by the Health Resources and Services Administration (HRSA). Race and ethnicity of a county population are self-reported by respondents to surveys and censuses by the US Census Bureau and incorporated into the Area Health Resources Files. For both NHSC clinicians and county populations, this study defined 4 categories of race and ethnicity, including Hispanic, non-Hispanic Black, non-Hispanic White, and non-Hispanic other (American Indian and Alaska Native, Asian, Native Hawaiian and Other Pacific Islander, and other race). Source: Authors’ analysis of 2003 to 2019 NHSC Field Strength data, HRSA, and US Census Bureau data.

Figure 2 illustrates the temporal trends in the national-level community representativeness ratio across the 3 HPSA designation types. Initially, NHSC Hispanic clinicians were overrepresented relative to Hispanic individuals in the population. For example, in 2003 the proportion of Hispanic NHSC primary care and dental care clinicians was 3.5 times higher than the proportion of Hispanic individuals among the population of primary care and dental care HPSAs. Similarly, the proportion of mental health care Hispanic clinicians in 2003 exceeded the proportion of Hispanic individuals in mental health care HPSAs by 2.5 times. However, the community representativeness ratio for Hispanic individuals declined consistently over time, reaching parity in 2011 and transitioning from overrepresentation to underrepresentation since 2012. In contrast, the community representativeness ratios for White and Black individuals were consistently below parity during the 2003 to 2008 period, indicating their underrepresentation among NHSC clinicians compared with the population. However, following the 2009 NHSC expansion, the community representativeness ratios steadily increased for White and Black individuals. By 2011, the percentage of Black NHSC clinicians in primary care and dental health care became equal to the percentage of Black individuals in the population, and the community representativeness ratios remained at or above parity thereafter. Black mental health clinicians remained below parity but made significant gains in clinician representation relative to population, with the community representativeness ratio for this group increasing from nearly 0 in 2003 to 0.77 in 2019.

eFigure 5 in Supplement 1 illustrates the temporal trends in the county-level community representativeness ratio across the 3 HPSA designation types. These county-level trends largely mirror the national-level trends across all race and ethnicity and clinician specialty groups. Notably, the county-level community representativeness ratio for White individuals is lower than the national-level ratio. This may be because counties with 0 practitioners weight the average of the ratio toward 0. As discussed in the Methods section, county-level community representativeness measures suffer from overinflation when NHSC clinician totals per county are small. This is reflected in the very high ratio before 2009 for Hispanic clinicians. County-level analysis enables us to statistically assess distance from parity, showing that Black individuals in the primary care field were significantly above parity in 2017 and 2018 (community representativeness ratio, 1.52; 95% CI, 1.06-1.98; and 1.50; 95% CI, 1.06-1.94, respectively), and that other race individuals significantly exceeded parity from 2010 through 2014 (community representativeness ratio, 2.08; 95% CI, 1.74-2.42 in 2010, declining to 1.53; 95% CI, 1.19-1.86 in 2014).

**Figure 3.**
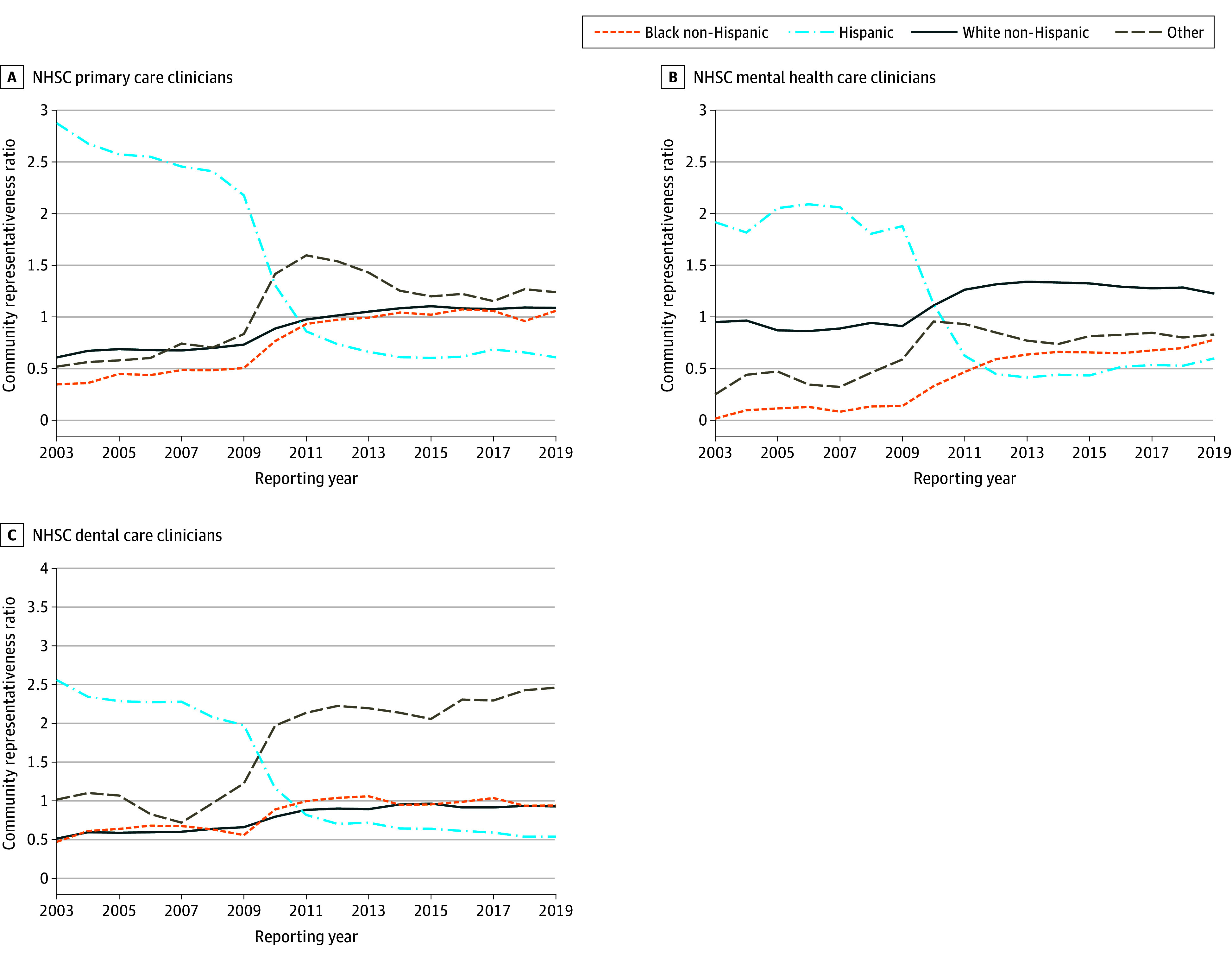
Community Representativeness Ratio for Counties With at Least 1 National Health Service Corps (NHSC) Clinician, by Clinician Specialty and Race and Ethnicity The community representativeness ratio measure was defined as the ratio of the proportion of NHSC clinicians of a given race and ethnicity divided by the proportion of the population of shortage counties with at least 1 NHSC clinician who identified as the same race and ethnicity. Diversity indices were calculated for primary care, mental health care, and dental care NHSC clinicians relative to the population of primary care, mental health care, and dental care health professional shortage areas with more than 1 clinician. Race and ethnicity of NHSC clinicians are self-reported in the NHSC clinician database compiled by the Health Resources and Services Administration (HRSA). Race and ethnicity of a county population are self-reported by respondents to surveys and censuses by the US Census Bureau and incorporated into the Area Health Resources Files. For both NHSC clinicians and county populations, this study defined 4 categories of race and ethnicity, including non-Hispanic White, non-Hispanic Black, Hispanic, and non-Hispanic other (American Indian and Alaska Native, Asian, Native Hawaiian and Other Pacific Islander, and other race). Source: Authors’ analysis of 2003 to 2019 NHSC Field Strength data, HRSA, and US Census Bureau data.

As indicated in eFigure 6 in Supplement 1, the direction and magnitude of change in the clinician-patient ratio are similar to the community representativeness ratio. Figure 3 illustrates the trends in the aggregate community representativeness ratio for counties with at least 1 primary, mental health, or dental care NHSC clinician. These trends mirror the trends in community representativeness ratio for all HPSA-designated counties. The trends in the national community representativeness ratio for NHSC physicians vs other NHSC clinicians (see eFigure 2 and eFigure 3 in Supplement 1) align with the trends among all NHSC clinicians discussed above.

Trends in the aggregate community representativeness ratio for counties consistently designated as whole county HPSA are presented in eFigure 4 in Supplement 1. Notably, in these HPSAs, the community representativeness ratio for Hispanic individuals in primary care remained at parity after 2011, in contrast to the decline observed across all HPSAs. Additionally, the community representativeness ratio for Black individuals in mental health care exceeded parity after 2012, in contrast to not reaching parity when all HPSAs were examined.

## Discussion

Improving racial and ethnic diversity of the health workforce is essential for increasing access to culturally competent care for patients in underserved areas.^31^ While the NHSC expansion since 2009 has increased the availability of clinicians in underserved communities, it is important to assess if the racial and ethnic characteristics of the NHSC workforce are becoming more concordant with those of the residents in their practice areas. To our knowledge, this is the first study to assess the long-term trends in the NHSC clinician diversity and the racial and ethnic concordance between NHSC clinicians and their potential patient population. We found that the NHSC workforce growth since 2009 was due to an increase in White NHSC clinicians, while the number of Hispanic NHSC clinicians remained stagnant and their proportional representation declined after 2009. Several factors may have contributed to the lack of growth among Hispanic NHSC clinicians after 2009. First, financial constraints and the burden of student loan debt may disproportionately affect minority NHSC clinicians for whom scholarships would be much more attractive than loan repayment as enrollment incentives.^32,33,34^ And in fact, we found that the number of Hispanic clinicians enrolled in the NHSC scholarship program declined over time. Second, other barriers to recruitment such as disparities in access to premedical and medical education limit the influx of Hispanic individuals into the medical profession^35^ and may account for the relatively stagnant number of Hispanic clinicians among the NHSC workforce. The sustained increase of Black NHSC clinicians following the 2009 NHSC expansion may be explained by the extremely low number of Black NHSC clinicians before 2009—a yearly average of 190 Black clinicians, as opposed to 1681 for Hispanic clinicians.

Before the 2009 NHSC expansion, White and Black NHSC clinicians were underrepresented relative to their respective populations, while Hispanic NHSC clinicians were overrepresented. The increase in the NHSC workforce since 2009 led to a closer alignment between White and Black NHSC clinicians and their respective populations. However, the representation of Hispanic NHSC clinicians declined to below-population levels after 2009. This proportional decline in Hispanic NHSC clinicians is particularly worrisome considering the projected growth of the Hispanic population, greater likelihood of linguistic discordance, and their increasing health care needs.^36,37^ Despite this finding, we note that the proportion of racial and ethnic minority clinicians, including Hispanic clinicians, among the NHSC workforce is higher relative to their share in the national workforce.^38^

Although there was an overrepresentation of Hispanic NHSC clinicians before 2009, there were very few NHSC professionals overall, making it unlikely that there was a significant discordance in clinical visits. After 2009, the growth of Hispanic NHSC clinicians lagged behind other clinician groups, preventing them from keeping up with the rapid growth of the Hispanic population across the country, including underserved areas. To address the issue of under-representation of NHSC Hispanic clinicians, policymakers need to take multiple actions, such as targeting Hispanic clinicians in future NHSC recruitment efforts, providing more NHSC scholarships to Hispanic students, and enrolling more Hispanic students in medical and health professional training programs.

The recent Supreme Court ruling to eliminate affirmative action for college admission is expected to have negative impacts on diversity in medical education.^39,40^ Therefore, it is important for researchers to monitor how this ruling may affect the growth of underrepresented clinicians, particularly Black, Hispanic, and American Indian or Alaska Native clinicians, in the overall health workforce and the NHSC program.

It is important to acknowledge that patient-clinician concordance extends beyond racial and ethnic factors. Other sociocultural factors, such as language proficiency, cultural competence, and shared experiences may mediate the association between racial and ethnic concordance and better health outcomes.^41^ Future research could incorporate these dimensions to gain a more comprehensive understanding of whether the NHSC program improved patient-clinician relationships across racial and ethnic lines. Future research might also examine whether greater racial, ethnic, and linguistic concordance between clinicians and patients in HPSAs reduces health care disparities.

### Limitations

This study has several limitations. First, we lack patient-level race and ethnicity data and instead provide a county-level assessment of patient-clinician concordance, not capturing the intricacies of individual-level patient-clinician dynamics. Nonetheless, our use of the community representativeness ratio at the county level is consistent with prior studies.^28,29^

Second, we combined the following groups into non-Hispanic other—Asian, American Indian or Alaskan Native, and Native Hawaiian or Other Pacific Islander. We were not able to analyze them separately due to low sample size.

Third, our analyses do not examine the diversity (or lack thereof) of non-NHSC clinicians serving patients in HPSA counties or compare clinician diversity between HPSA and non-HPSA counties. These diversity-related issues remain important topics for future research. Additionally, although the community representativeness ratio has been well established in the literature, it uses race and ethnicity as a single factor in assessing concordance, not encompassing other important elements of patient-clinician interactions, such as language proficiency, cultural competence, or shared experiences.

## Conclusion

Persistent shortages of health workforce pose a daunting challenge for the US health care system, and the NHSC program has played a critical role in attracting clinicians to underserved areas. We found that the increased number of NHSC clinicians since 2009 were associated with improved patient-clinician concordance for White individuals and Black individuals. Hispanic NHSC clinicians, however, became underrepresented relative to population. Our findings have important implications for the ongoing NHSC expansion given that racial and ethnic patient-clinician concordance has been recognized as an important factor for effective and inclusive care delivery.

## Notes

Supplement 1. **eTable. **Classification of NHSC Clinicians Into 3 Fields (Primary Care, Mental Health Care, and Dental Care) and 2 Groups (Physicians and Other Clinicians) **eFigure 1. **Number of NHSC Physicians by Race and Ethnicity, Specialty, and NHSC Program Type **eFigure 2. **Number of NHSC Physicians by Race and Ethnicity and Specialty **eFigure 3. **Number of NHSC Nonphysician Clinicians by Race and Ethnicity and Clinician Specialty **eFigure 4. **Community Representativeness Ratio in Always Whole Shortage County HPSAs, by Clinician Specialty and Race and Ethnicity **eFigure 5. **County-Level Community Representativeness Ratio, by Clinician Specialty and Race and Ethnicity **eFigure 6. **County-Level Clinician-Population Ratio by Clinician Specialty and Race and Ethnicity

Supplement 2.
Data Sharing Statement
