# Supplementary material for: Racial and Ethnic Concordance Between National Health Service Corps Clinicians and Underserved Populations
Source: JAMA Netw Open. 2024 Mar 20;7(3):e242961. doi: 10.1001/jamanetworkopen.2024.2961 (PMC10955390; doi:10.1001/jamanetworkopen.2024.2961)
Supplement: Supplement 1. — eTable. Classification of NHSC Clinicians Into 3 Fields (Primary Care, Mental Health Care, and Dental Care) and 2 Groups (Physicians and Other Clinicians) eFigure 1. Number of NHSC Physicians by Race and Ethnicity, Specialty, and NHSC Program Type eFigure 2. Number of NHSC Physicians by Race and Ethnicity and Specialty eFigure 3. Number of NHSC Nonphysician Clinicians by Race and Ethnicity and Clinician Specialty eFigure 4. Community Representativeness Ratio in Always Whole Shortage County HPSAs, by Clinician Specialty and Race and Ethnicity eFigure 5. County-Level Community Representativeness Ratio, by Clinician Specialty and Race and Ethnicity eFigure 6. County-Level Clinician-Population Ratio by Clinician Specialty and Race and Ethnicity [file jamanetwopen-e242961-s001.pdf]

Supplemental Online Content

Baker O, Horvitz-Lennon M, Yu H. Racial and ethnic concordance between National Health Service Corps clinicians and underserved populations. *JAMA Netw Open*. 2024;7(3):e242961.
doi:10.1001/jamanetworkopen.2024.2961

eTable. Classification of NHSC Clinicians Into 3 Fields (Primary Care, Mental Health Care, and Dental Care) and 2 Groups (Physicians and Other Clinicians)

eFigure 1. Number of NHSC Physicians by Race and Ethnicity, Specialty, and NHSC Program Type

eFigure 2. Number of NHSC Physicians by Race and Ethnicity and Specialty

eFigure 3. Number of NHSC Nonphysician Clinicians by Race and Ethnicity and Clinician Specialty

eFigure 4. Community Representativeness Ratio in Always Whole Shortage County HPSAs, by Clinician Specialty and Race and Ethnicity

eFigure 5. County-Level Community Representativeness Ratio, by Clinician Specialty and Race and Ethnicity

eFigure 6. County-Level Clinician-Population Ratio by Clinician Specialty and Race and Ethnicity

This supplemental material has been provided by the authors to give readers additional information about their work.

© 2024 Baker O et al. *JAMA Network Open.*

eTable. Classification of NHSC Clinicians Into 3 Fields (Primary Care, Mental Health Care, and Dental Care) and 2 Groups (Physicians and Other Clinicians)

Field	Physician vs non-Physician Clinicians	Clinician Type	Specialty
Primary Care	Physician	Allopathic Physician	Family Practice
Primary Care	Physician	Allopathic Physician	Family Practice - Geriatrics
Primary Care	Physician	Allopathic Physician	Family Practice w/OB
Primary Care	Physician	Allopathic Physician	Internal Medicine
Primary Care	Physician	Allopathic Physician	Internal Medicine - Geriatrics
Primary Care	Physician	Allopathic Physician	None
Primary Care	Physician	Allopathic Physician	OB/GYN
Primary Care	Physician	Allopathic Physician	Pediatrics
Primary Care	Physician	Osteopathic Physician	Family Practice
Primary Care	Physician	Osteopathic Physician	Family Practice - Geriatrics
Primary Care	Physician	Osteopathic Physician	Family Practice w/OB
Primary Care	Physician	Osteopathic Physician	General Practice
Primary Care	Physician	Osteopathic Physician	Internal Medicine
Primary Care	Physician	Osteopathic Physician	Internal Medicine - Geriatrics
Primary Care	Physician	Osteopathic Physician	OB/GYN
Primary Care	Physician	Osteopathic Physician	Pediatrics
Primary Care	non- Physician	Certified Nurse Midwife	None
Primary Care	non- Physician	Chiropractor	None
Primary Care	non- Physician	Clinical Nurse Specialist	None
Primary Care	non- Physician	Nurse Practitioner	Adult

© 2024 Baker O et al. *JAMA Network Open.*

Primary Care	non- Physician	Nurse Practitioner	Family Practice
Primary Care	non- Physician	Nurse Practitioner	Geriatrics
Primary Care	non- Physician	Nurse Practitioner	None
Primary Care	non- Physician	Nurse Practitioner	Pediatrics
Primary Care	non- Physician	Nurse Practitioner	Women's Health
Primary Care	non- Physician	Pharmacist	None
Primary Care	non- Physician	Physician Assistant	Adult
Primary Care	non- Physician	Physician Assistant	Family Practice
Primary Care	non- Physician	Registered Nurse	None
Primary Care	non- Physician	Registered Nurse Anesthetist	None
Primary Care	non- Physician	Physician Assistant	None
Primary Care	non- Physician	Physician Assistant	Pediatrics
Primary Care	non- Physician	Physician Assistant	Women's Health
Mental Health	Physician	Allopathic Physician	Psychiatry
Mental Health	Physician	Allopathic Physician	Psychiatry - Geriatrics
Mental Health	Physician	Osteopathic Physician	Psychiatry
Mental Health	non- Physician	Health Service Psychologist	None
Mental Health	non- Physician	Licensed Clinical Social Worker	None
Mental Health	non- Physician	Licensed Professional Counselor	None
Mental Health	non- Physician	Marriage and Family Therapist	None
Mental Health	non- Physician	Nurse Practitioner	Psychiatry
Mental Health	non- Physician	Physician Assistant	Psychiatry
Mental Health	non- Physician	Psychiatric Nurse Specialist	Adult

© 2024 Baker O et al. *JAMA Network Open.*

Mental Health	non- Physician	Psychiatric Nurse Specialist	None
Mental Health	non- Physician	Psychiatric Nurse Specialist	Pediatrics
Mental Health	non- Physician	Substance Use Disorder Counselor	None
Dentist	Physician	Dentist	General Practice
Dentist	Physician	Dentist	Geriatrics
Dentist	Physician	Dentist	None
Dentist	Physician	Dentist	Pediatrics
Dentist	Physician	Dentist	Public Health Dentistry
Dentist	non- Physician	Registered Dental Hygienist	None

© 2024 Baker O et al. *JAMA Network Open.*

eFigure 1. Number of NHSC Physicians by Race and Ethnicity, Specialty, and NHSC Program Type.
SOURCE: Authors' analysis of NHSC Field Strength data, FY 2003 – FY 2019.
NOTES: Vertical line denotes the 2009 NHSC expansion. Race and ethnicity of NHSC clinicians are self-reported in the NHSC clinician database compiled by HRSA.

Figure 1a
Caption: NHSC Primary Care Physicians in the Loan Repayment Program.

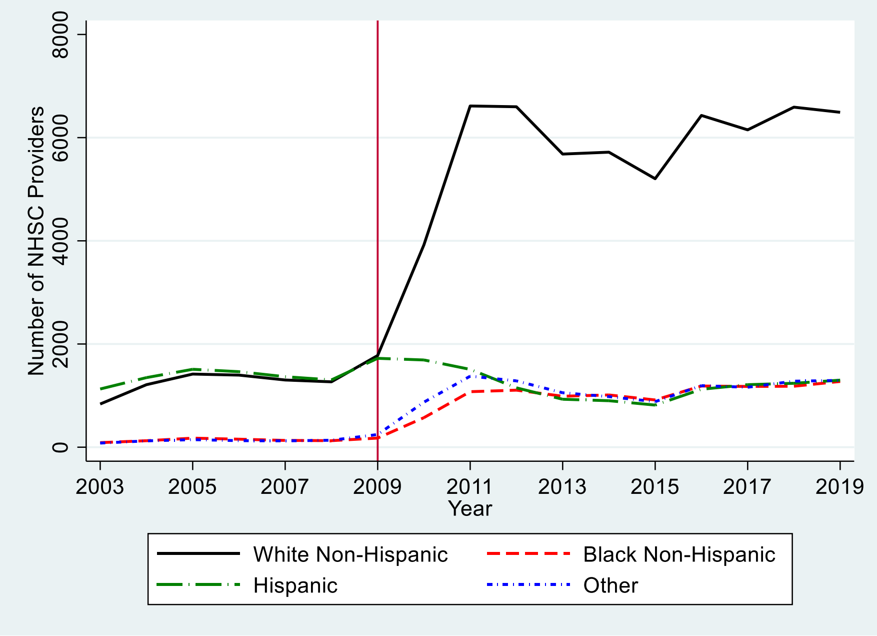

Figure 1b
Caption: NHSC Primary Care Physicians in the Scholarship Program.

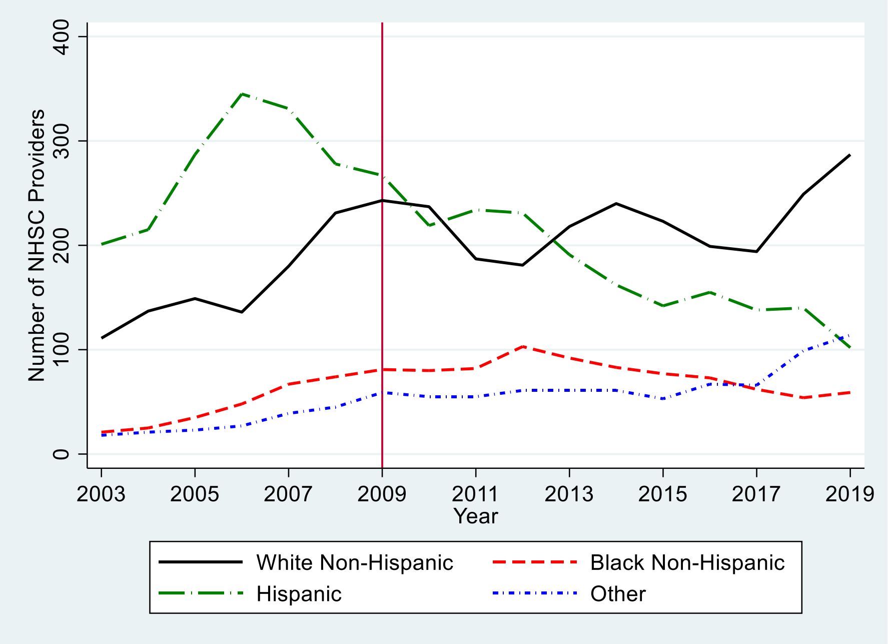

© 2024 Baker O et al. *JAMA Network Open.*

eFigure 2. Number of NHSC Physicians by Race and Ethnicity and Specialty.
SOURCE: Authors' analysis of NHSC Field Strength data, FY 2003 – FY 2019.
NOTES: Vertical line denotes the 2009 NHSC expansion. Race and ethnicity of NHSC clinicians are self-reported in the NHSC clinician database compiled by HRSA.

Figure 2a
Caption: NHSC Primary Care Physicians.

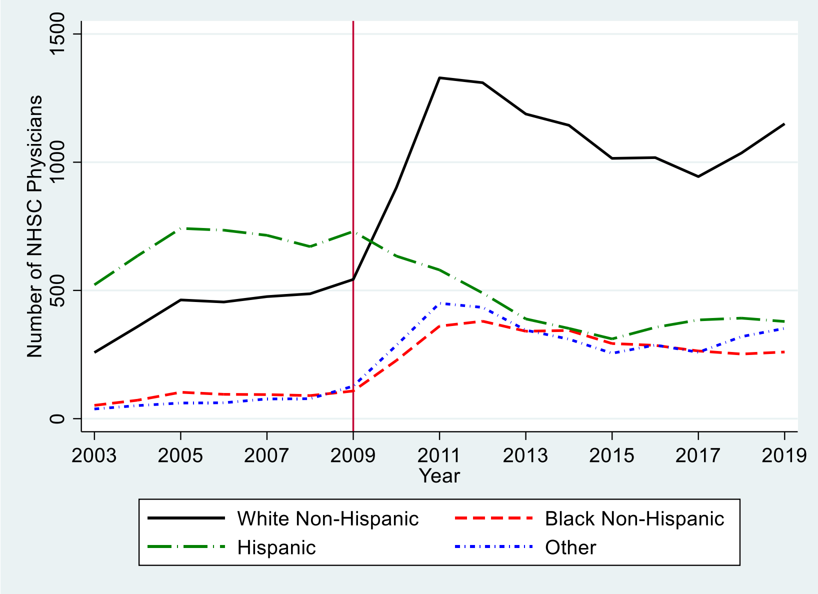

Figure 2b
Caption: NHSC Mental Health Care Psychiatrists.

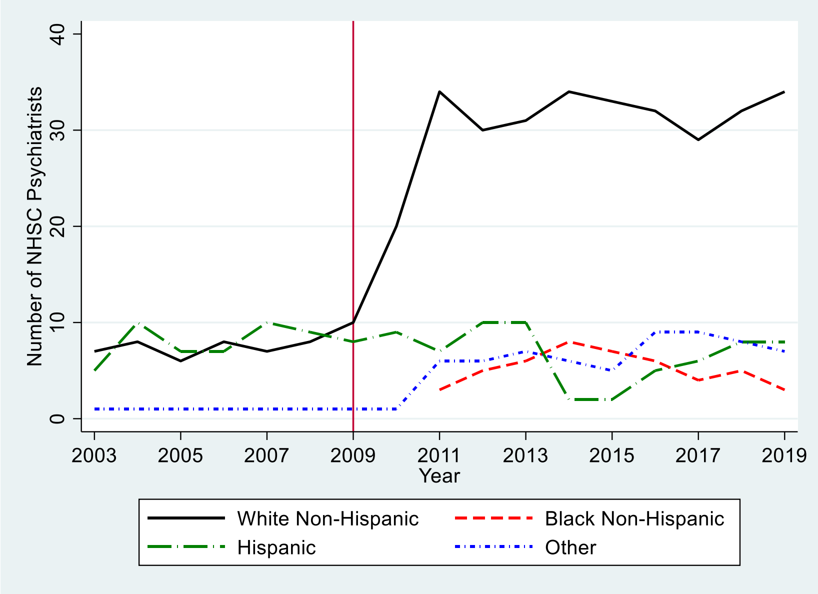

© 2024 Baker O et al. *JAMA Network Open.*

Figure 2c

Caption: NHSC Dentists.

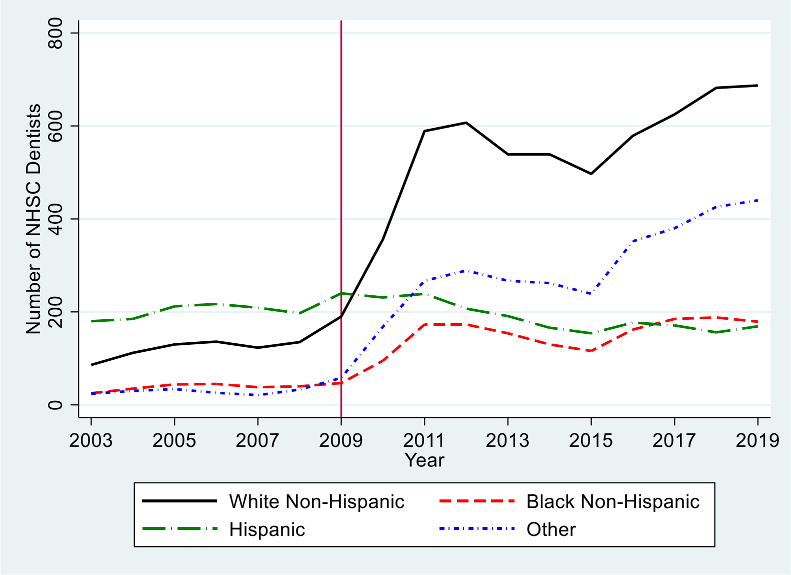

© 2024 Baker O et al. *JAMA Network Open.*

eFigure 3. Number of NHSC Nonphysician Clinicians by Race and Ethnicity and Clinician Specialty.
SOURCE: Authors' analysis of NHSC Field Strength data, FY 2003 – FY 2019.
NOTES: Vertical line denotes the 2009 NHSC expansion. Race and ethnicity of NHSC clinicians are self-reported in the NHSC clinician database compiled by HRSA.

Figure 3a
Caption: NHSC Primary Care Non-Physician Clinicians.

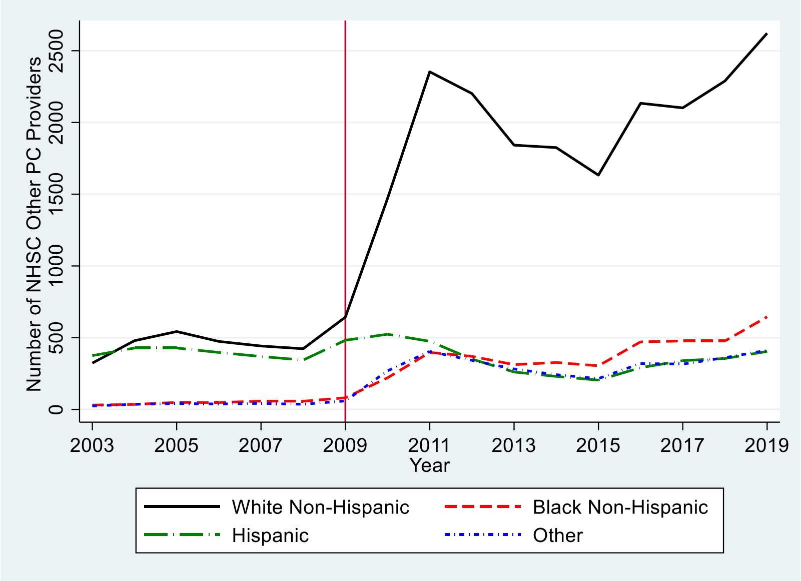

Figure 3b
Caption: NHSC Mental Health Care Non-Physician Clinicians.

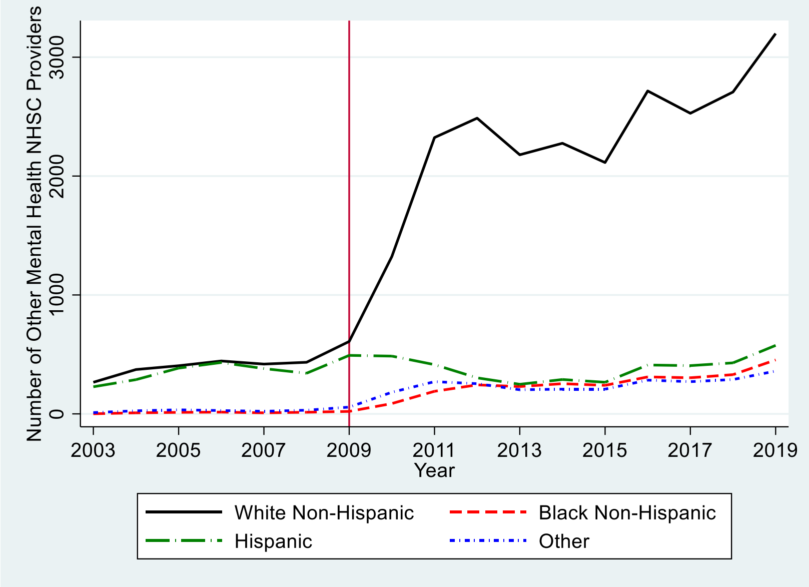

© 2024 Baker O et al. *JAMA Network Open.*

Figure 3c

Caption: NHSC Dental Care Non-Physician Clinicians.

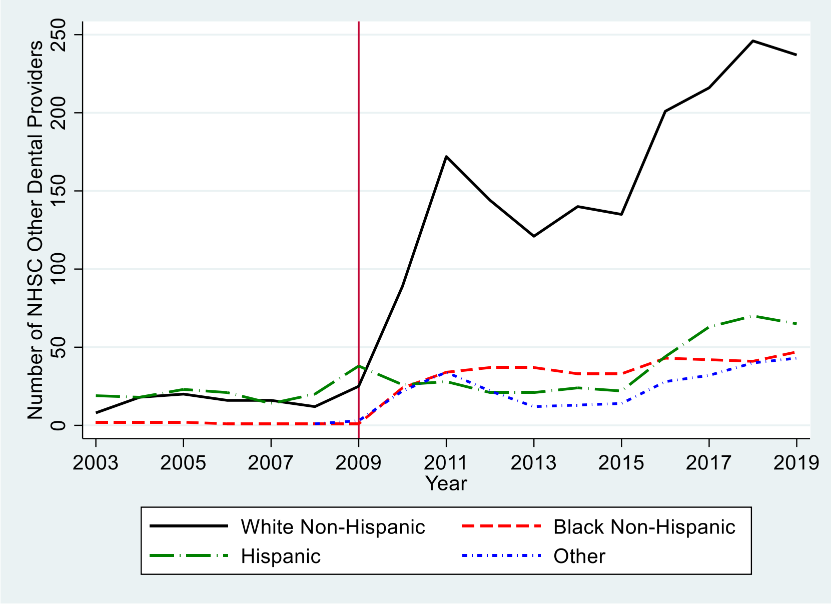

© 2024 Baker O et al. *JAMA Network Open.*

eFigure 4. Community Representativeness Ratio in Always Whole Shortage County HPSAs, by Clinician Specialty and Race and Ethnicity.

SOURCE: Authors' analysis of 2003-2019 NHSC Field Strength data, HRSA and US Census data.

NOTES: Horizontal line with Community representativeness ratio equal to 1 denotes parity. Race and ethnicity of NHSC clinicians are self-reported in the NHSC clinician database compiled by HRSA. Race and ethnicity of a county population are self-reported by respondents to surveys and censuses by the United States Census Bureau and incorporated into the Area Health Resources Files. For both NHSC clinicians and county populations, this study defined four categories of race and ethnicity, including non-Hispanic White, non-Hispanic Black, Hispanic, and non-Hispanic Other (American Indian and Alaska Native, Asian, Native Hawaiian and Other Pacific Islander, and Other Race).

Figure 4a

Caption: NHSC Primary Care Clinicians.

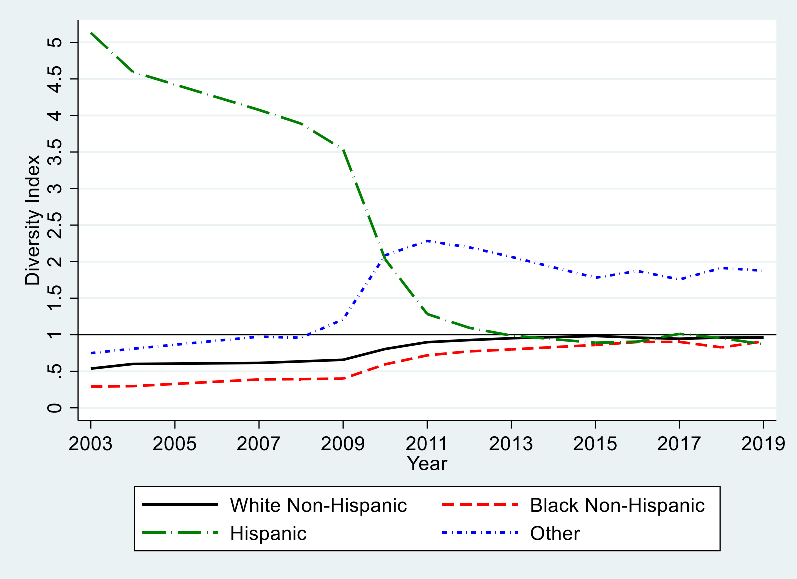

Figure 4b

Caption: NHSC Mental Health Care Clinicians.

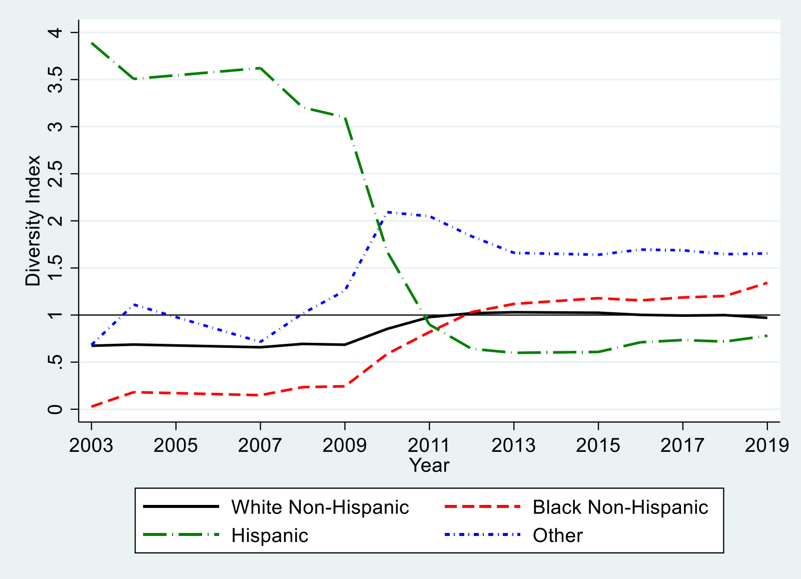

© 2024 Baker O et al. *JAMA Network Open.*

Figure 4c

Caption: NHSC Dental Care Clinicians.

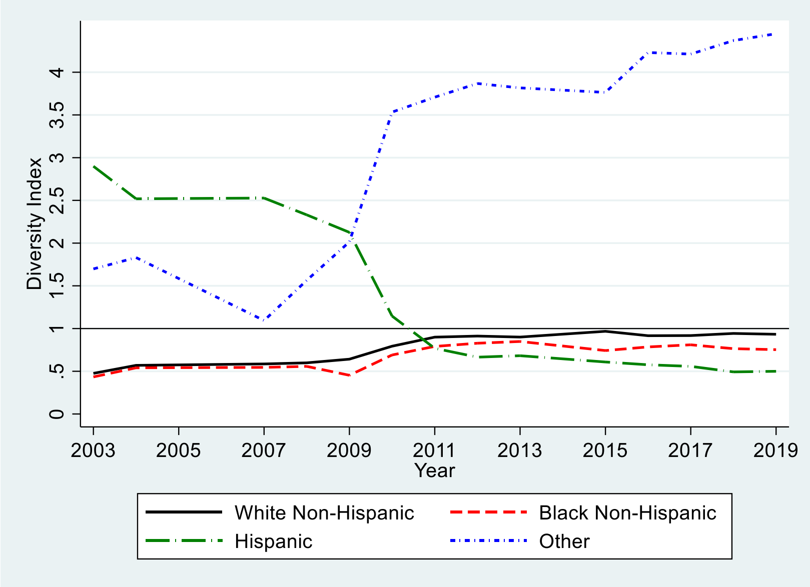

© 2024 Baker O et al. *JAMA Network Open.*

eFigure 5. County-Level Community Representativeness Ratio, by Clinician Specialty and Race and Ethnicity.
SOURCE: Authors' analysis of 2003-2019 NHSC Field Strength data, HRSA and US Census data.
NOTES: Means of community representativeness ratio (dots) and 95% confidence intervals (bars). Unadjusted means were calculated and plotted using Stata's -margins- command. Community representativeness ratio equal to 1 denotes parity. Race and ethnicity of a county population are self-reported by respondents to surveys and censuses by the United States Census Bureau and incorporated into the Area Health Resources Files. For both NHSC clinicians and county populations, this study defined four categories of race and ethnicity, including non-Hispanic White, non-Hispanic Black, Hispanic, and non-Hispanic Other (American Indian and Alaska Native, Asian, Native Hawaiian and Other Pacific Islander, and Other Race).

Figure 5a
Caption: Primary Care

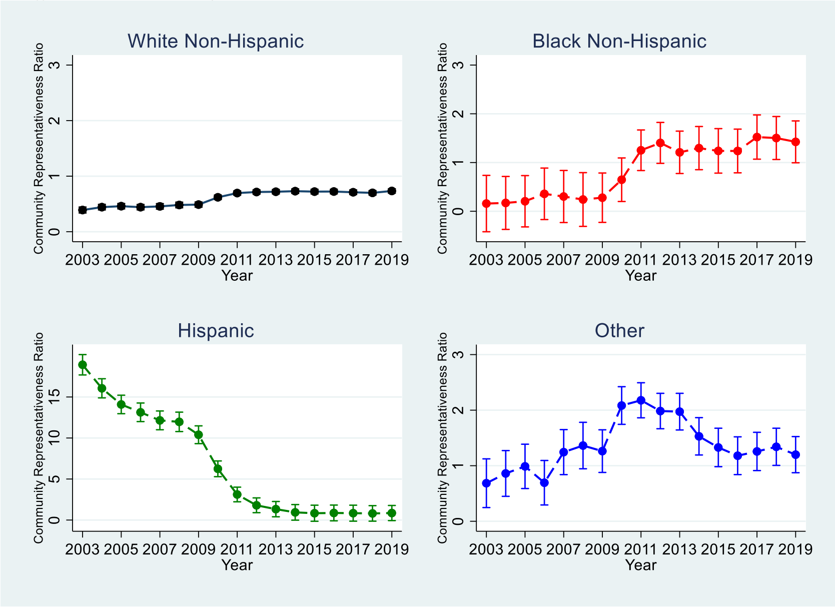

Figure 5b
Caption: Mental Health Care

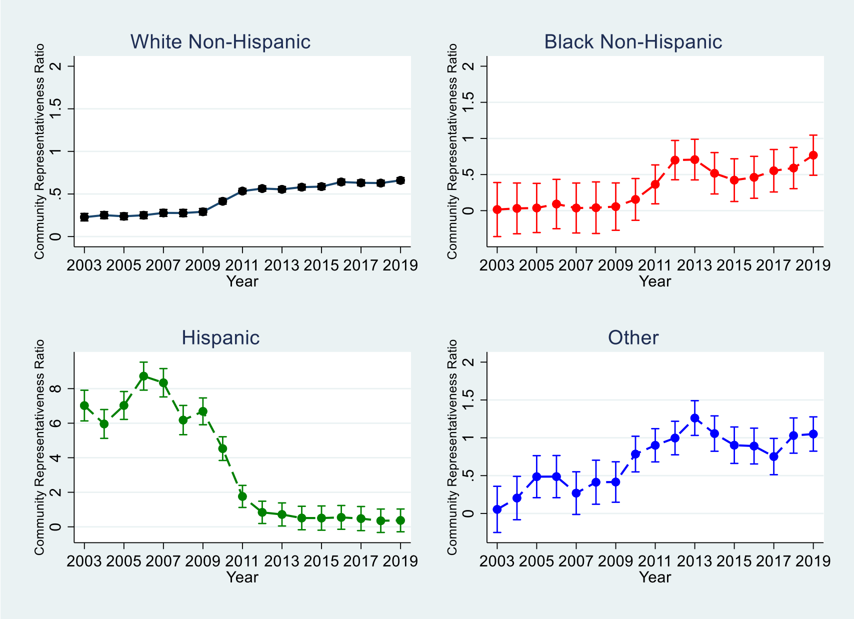

© 2024 Baker O et al. *JAMA Network Open.*

Figure 5c

Caption: Dental Care

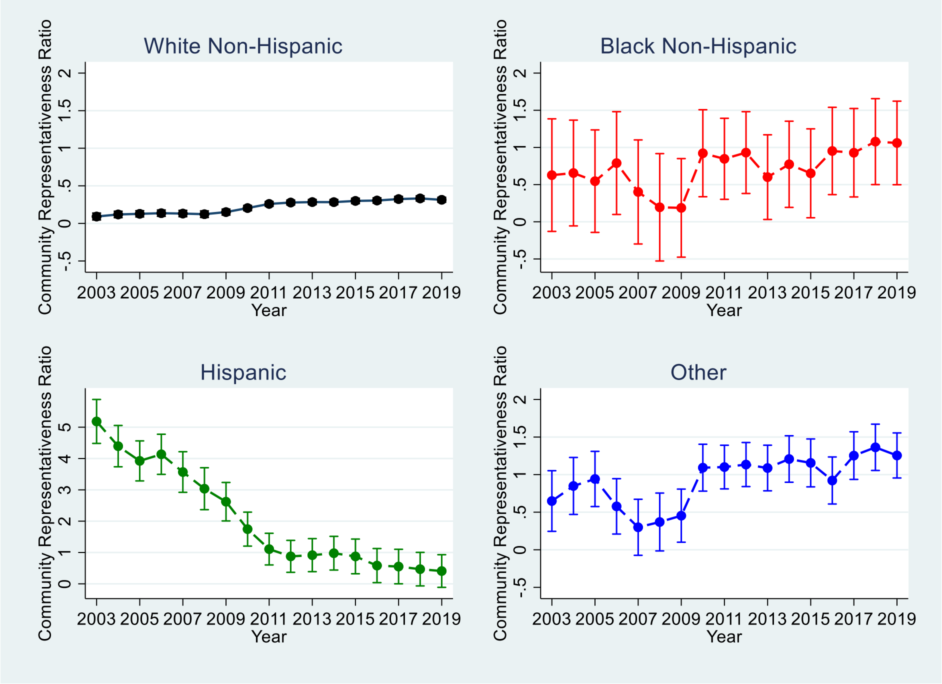

© 2024 Baker O et al. *JAMA Network Open.*

eFigure 6. County-Level Clinician-Population Ratio by Clinician Specialty and Race and Ethnicity.
SOURCE: Authors' analysis of 2003-2019 NHSC Field Strength data, HRSA and US Census data.
NOTES: Means of community representativeness ratio (dots) and 95% confidence intervals (bars). Unadjusted means were calculated and plotted using Stata's -margins- command. Community representativeness ratio equal to 1 denotes parity. Race and ethnicity of a county population are self-reported by respondents to surveys and censuses by the United States Census Bureau and incorporated into the Area Health Resources Files. For both NHSC clinicians and county populations, this study defined four categories of race and ethnicity, including non-Hispanic White, non-Hispanic Black, Hispanic, and non-Hispanic Other (American Indian and Alaska Native, Asian, Native Hawaiian and Other Pacific Islander, and Other Race).

Figure 6a

Caption: Primary Care

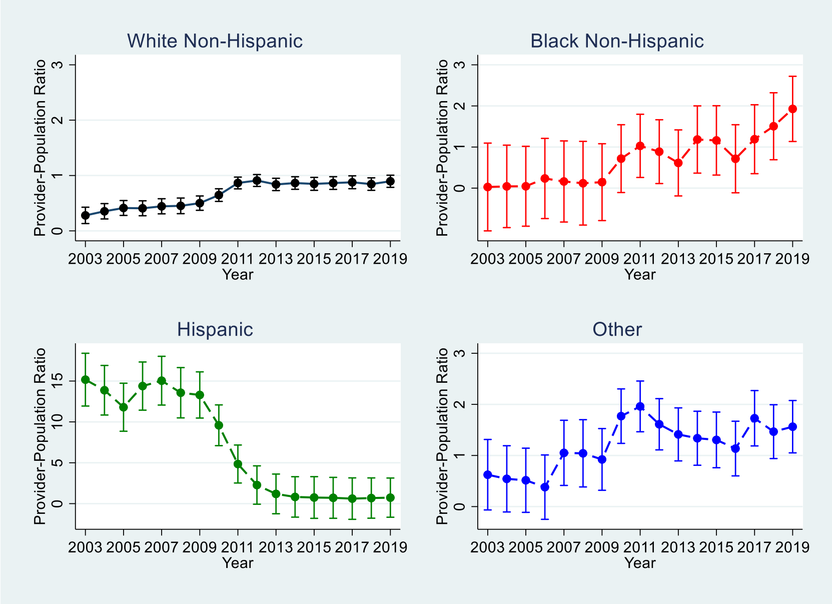

Figure 6b

Caption: Mental Health Care

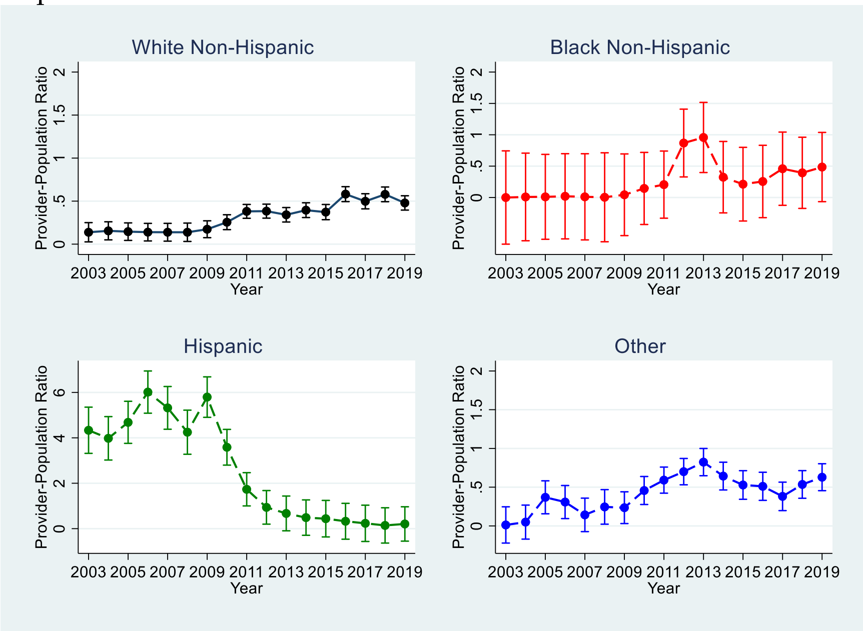

© 2024 Baker O et al. *JAMA Network Open.*

Figure 6c

Caption: Dental Care

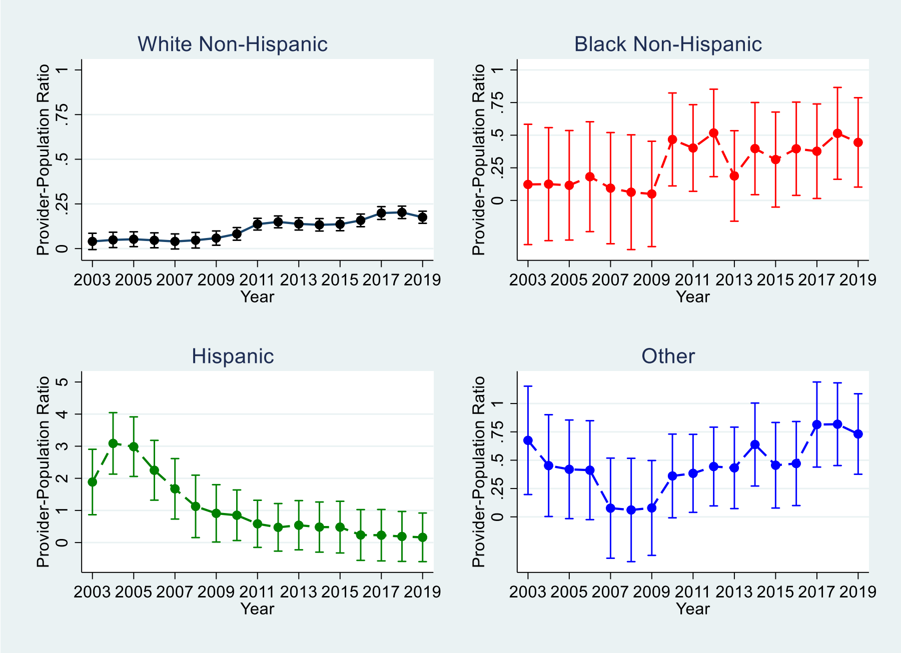

© 2024 Baker O et al. *JAMA Network Open.*
