# Supplementary material for: Racial and Ethnic Concordance Between National Health Service Corps Clinicians and Underserved Populations
Source: JAMA Netw Open. 2024 Mar 20;7(3):e242961. doi: 10.1001/jamanetworkopen.2024.2961 (PMC10955390; doi:10.1001/jamanetworkopen.2024.2961)
Supplement: Supplement 2. — Data Sharing Statement [file jamanetwopen-e242961-s002.pdf]

Data Sharing Statement

Baker. Racial and Ethnic Concordance Between National Health Service Corps Clinicians and Underserved Populations. *JAMA Netw Open*. Published March 20, 2024. doi:10.1001/jamanetworkopen.2024.2961

Data
Data available: No

Additional Information
Explanation for why data not available: Both the AHRF and the NHSC field strength data are publicly available at https://data.hrsa.gov/data/downloadand
